# The Arabidopsis L-Type Amino Acid Transporter 5 (LAT5/PUT5) Is Expressed in the Phloem and Alters Seed Nitrogen Content When Knocked Out

**DOI:** 10.3390/plants9111519

**Published:** 2020-11-09

**Authors:** Rowshon A. Begam, Jayne D’Entremont, Allen Good

**Affiliations:** Department of Biological Sciences, University of Alberta, Edmonton, AB T6G 2E9, Canada; dentremo@ualberta.ca (J.D.); allen.good@ualberta.ca (A.G.)

**Keywords:** LAT5, PUT5, L-type amino acid transporter, amino acids, LAT, polyamine uptake transporter, nitrogen, Arabidopsis, amino acid translocation, seed nitrogen

## Abstract

The Arabidopsis L-type Amino Acid Transporter-5 (LAT5; At3g19553) was recently studied for its role in developmental responses such as flowering and senescence, under an assumption that it is a polyamine uptake transporter (PUT5). The LATs in Arabidopsis have a wide range of substrates, including amino acids and polyamines. This report extensively studied the organ and tissue-specific expression of the LAT5/PUT5 and investigated its role in mediating amino acid transport. Organ-specific quantitative RT-PCR detected LAT5/PUT5 transcripts in all organs with a relatively higher abundance in the leaves. Tissue-specific expression analysis identified GUS activity in the phloem under the LAT5/PUT5 promoter. In silico analysis identified both amino acid transporter and antiporter domains conserved in the LAT5/PUT5 protein. The physiological role of the LAT5/PUT5 was studied through analyzing a mutant line, *lat5-1*, under various growth conditions. The mutant *lat5-1* seedlings showed increased sensitivity to exogenous leucine in Murashige and Skoog growth medium. In soil, the *lat5-1* showed reduced leaf growth and altered nitrogen content in the seeds. In planta radio-labelled leucine uptake studies showed increased accumulation of leucine in the *lat5-1* plants compared to the wild type when treated in the dark prior to the isotopic feeding. These studies suggest that LAT5/PUT5 plays a role in mediating amino acid transport.

## 1. Introduction

Plants take up nitrogen in the forms of NO_3_^−^, NH_4_^+^, amino acids, and peptides. Nitrogen, taken up as NO_3_^−^ or NH_4_^+^, is assimilated into amino acids via the Glutamine Synthetase/Glutamate Synthase pathway and moves from the source to sink tissues in the form of amino acids [1,2]. Thus, the functional characterization of plant amino acid transporters is important to elucidate plants’ ability to take up organic nitrogen from the soil and to understand the translocation and distribution of nitrogen within plants. Amino acids travel from the source to sink tissues through various membranes (plasma, chloroplast, mitochondria, tonoplast, and peroxisome) via facilitated or active transport by amino acid transporters. Plants with various types of source and sink tissues, as well as with various types of protein and non-protein amino acids, require a large number of amino acid transporters with export, import, antiport, or facilitator capacity. These transporters, as they facilitate the movement of amino acids through membranes, also regulate the distribution of amino-N in plants. Understanding the distribution of amino-N in plants thus requires understanding the tissue-specific distribution of amino acid transporters with their substrate affinity and transport mechanism.

While a large number of the annotated amino acid transporters in plants remain to be characterized, many of the characterized amino acid transporters were studied in heterologous unicellular systems such as mutant strains of *Saccharomyces cerevisiae* or oocytes of *Xenopus laevis* [3] (pp. 212–215). These relatively simple systems facilitate studying transporters for their transport mechanisms and substrate affinities under a controlled environment. However, the content and composition of amino acids in and out of a plant cell in a physiological environment is dynamic and differ from the experimental conditions provided in the heterologous uptake studies. Some amino acid transporters, such as the human LATs, require additional components for the tertiary folding and localization, and binding partners for the transport activity, which may not be available in a heterologous system [4,5,6]. Thus, in planta characterization of amino acid transporters is a preferential choice for understanding their biological importance. However, in the presence of over 60 amino acid transporters in Arabidopsis with possible redundant functions, it can be challenging to pinpoint the role of an amino acid transporter through single knockout mutant analysis [7]. This research investigated the role of the Arabidopsis L-type Amino acid Transporter-5 (LAT5; At3G19553) in physiological conditions and interpreted the findings with caution, keeping the shortcomings in consideration.

The Arabidopsis LAT family (LAT1-5), within the Amino acid Polyamine Choline superfamily, was initially classified based on a phylogenetic analysis that found their sequence similarity with the light chain subunits of the human L-type amino acid transport system [8]. Subsequently, the Arabidopsis LATs were re-classified as members of the proton–polyamine symporter (PHS) family [9]. The LATs in human function as heteromeric exchangers of large neutral amino acids at 1:1 stoichiometry [4,5,6,10]. However, the Arabidopsis LATs have been demonstrated to have roles in mediating the transport of a range of substrates including amino acid, polyamine, paraquat, and thiamine [11,12,13,14,15,16,17]. The LAT5 was previously studied for its role in developmental responses, such as flowering and senescence, where it was called PUT5 under the assumption that it is a polyamine uptake transporter [18]. While studies have yet to demonstrate its role in polyamine transport, one report mentioned that PUT5, when expressed in a mutant yeast strain deficient in spermidine transport, increased the sensitivity of the yeast cells to exogenous polyamines and paraquat, suggesting that PUT5 mediates polyamine transport [17]. In this report, we explored the organ and tissue-specific expression of the LAT5, studied its role in large neutral amino acid (leucine) transport in the native physiological environment, and termed it as LAT5/PUT5.

## 2. Results

### 2.1. In Silico Analysis Indicates Amino Acid Transporter and Antiporter Function for the LAT5/PUT5

**Figure 1:**
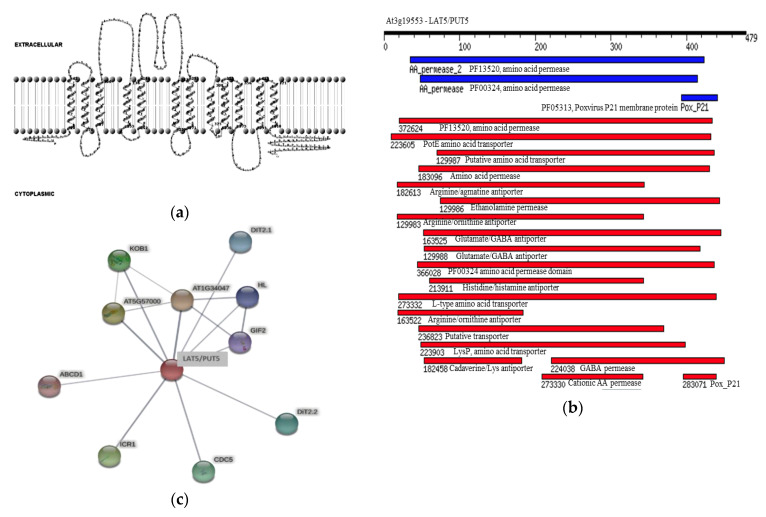
In silico characterization of the Arabidopsis LAT5/PUT5 protein. (**a**) A cartoon representation showing the 12 transmembrane domains in the LAT5/PUT5 protein with both c- and n- termini in the cytoplasmic side. (**b**) Domains conserved in the LAT5/PUT5 protein. The black bar shows the length of the amino acid sequence. The blue and red bars show the position and extent of the domains in LUT5/PUT5 protein. The blue bars show the conserved domains identified in the Pfam database and the red bars show the conserved domains identified in the NCBI conserved domain database. (**c**) Putative physical and functional associations of the LAT5/PUT5 with other proteins. The thickness of the gray line indicates the confidence level of the association between the two proteins. Thicker lines indicate higher confidence.

Based on in silico analyses, the Arabidopsis LAT5/PUT5 is an integral membrane protein, with a molecular weight of 52.8 kDa, a theoretical isoelectric point of 5.3, and 12 transmembrane domains with both the n- and c-termini in the cytoplasmic side (Figure 1a). In addition to the amino acid permease domain PF13520 reported to be conserved in all five Arabidopsis LATs [11], the LAT5/PUT5 putatively has PotE Amino Acid Transporter (COG0531), Amino Acid Permease (PRK11357), Arginine/Agmatine Antiporter (PRK10644), Arginine/Ornithine Antiporter (TIGR00905), and Glutamate/Gamma-Aminobutyrate Antiporter (TIGR00910) domains conserved in the amino acid sequence (Figure 1b). Human LATs have been demonstrated to form di-sulfide bridges with their glycoprotein subunits through a cysteine residue conserved between the third and fourth transmembrane domains [4]. Amino acid sequence alignment of the five Arabidopsis LATs identified a cysteine residue conserved near the c-termini of all five members (Table S1). However, the TMHMM prediction of transmembrane domains for the LAT5/PUT5 protein did not position the cysteine residue in any of the intra- or extra-cellular loops (Figure 1a). The conserved domain search identified a Pox_P21 (Pfam05313) domain near the c-terminus of the LAT5/PUT5 protein (Figure 1b). The protein interaction search using STRING identified several proteins such as Interactor of Constitutive active ROPs 1 (ICR1), ABC transporter D family member 1 (ABCD1), Dicarboxylate Transporter 2.1 (DiT2.1), and Dicarboxylate Transporter 2.2 (DiT2.2) to have possible functional associations with the LAT5/PUT5 protein (Figure 1c). The protein interaction search using the BioGRID identified the DEK domain-containing chromatin-associated protein (At4G26630; DEK3) and Cyclic Nucleotide-Gated Channel 13 (CNGC13) to have physical interaction with the LAT5/PUT5 protein. The protein associations identified using STRING have no experimental evidence. These were based on either co-mention of the two proteins in PubMed abstracts or their putative homologs were found interacting in other organisms. The interactions identified using BioGRID were based on experiments such as affinity capture-MS or Protein-Fragment Complementation Assay [19,20].

The Arabidopsis LAT5/PUT5 shares approximately 50% sequence identity with the rice Polyamine Uptake Transporter 1 (OsPUT1; Os02g47210) at the amino acid level. A PSI-BLASTp search in the NCBI non-redundant protein databank identified putative *Ricinus communis* neutral amino acid transporter (Accession: XP_002527494.1), *Populus trichocarpa* neutral amino acid transporters (Accession XP_002335345.1; XP_002300260.1; XP_002313902.1), and a *Medicago truncatula* neutral amino acid transporter (Accession: XP_003610625.1) that share more than 70% sequence similarity with the LAT5/PUT5 protein. A PSI-BLASTp search in the Protein Data Bank (http://www.pdb.org/) identified the *Escherichia coli* Arginine/Agmatine antiporter (AdiC; GI: 289526962) as the closest match with 23% sequence identity with the LAT5/PUT5.

### 2.2. The LAT5/PUT5 Is Expressed in All Organs with Specific Expression in the Phloem

**Figure 2:**
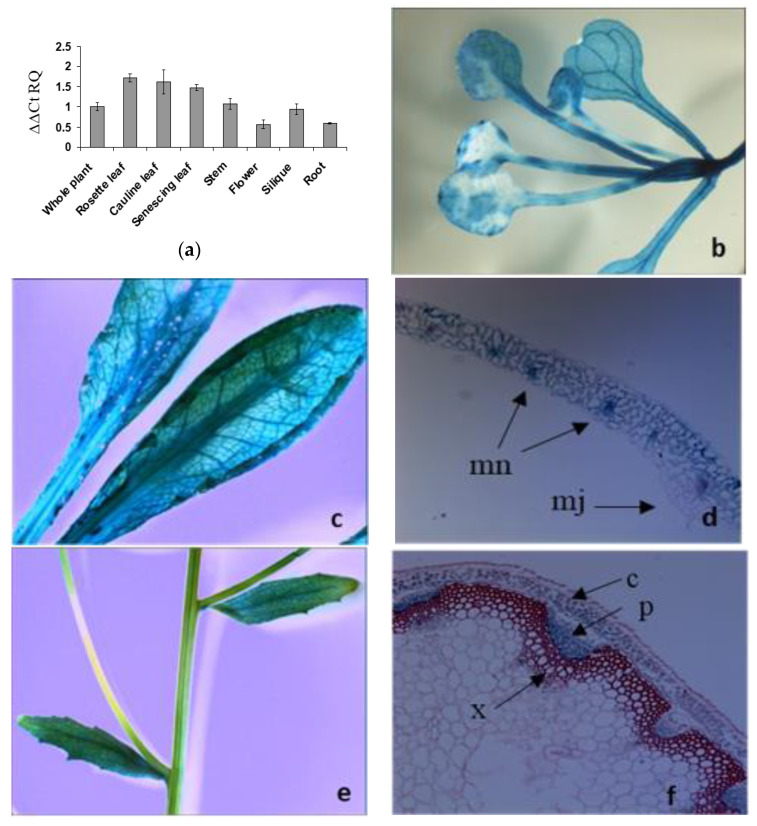

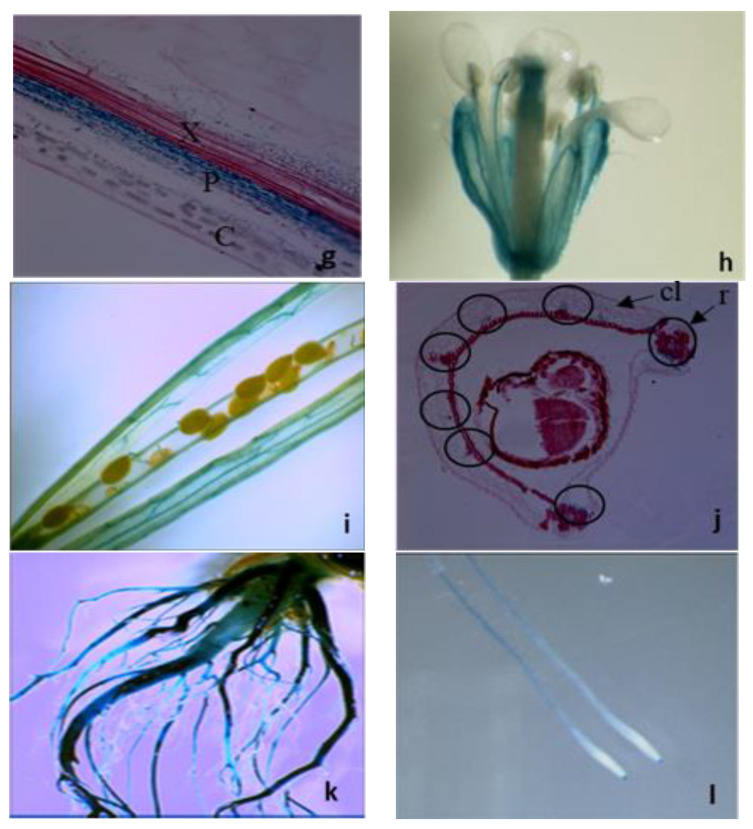
Organ- and tissue-specific expression analyses of the LAT5/PUT5. (**a**) Organ-specific quantitative RT–PCR detects the LAT5/PUT5 transcripts in all organs. The relative quantitation (RQ) was calculated using an average expression of the LAT5/PUT5 in whole plants as a calibrator and Actin2 as an endogenous control. The error bar represents the average difference between the highest and lowest RQ value of three biological replications each with three technical replications. (**b**–**j**) Tissue-specific expression of the LAT5/PUT5 based on GUS activity under the LAT5/PUT5 promoter. (**b**) GUS activity is present in the rosette leaf at the seeding stage; (**c**) Mature rosette leaf shows GUS stain; (**d**) A cross-section of the rosette leaf shows GUS stain in the mesophyll cells, concentrating more in the minor veins; (**e**) In mature stem, GUS stain is not visible on the stem surface; (**f**) A transverse section of a mature stem shows GUS stain in the phloem; (**g**) A longitudinal section of the stem shows GUS stain present in the phloem and absent in the xylem; (**h**) In the flower, GUS stain is present in the sepal and stamen filament; (**i**) In siliques, GUS stain is present along the vascular pattern in the fruit carpel; (**j**) Circles in the cross-section of a developing silique show the positions of the vascular tissues where GUS activity is present. GUS stain is present in the replum and the secondary vasculature in the carpel; (**k**) In mature plants, a GUS stain is present in the mature primary and lateral roots; (**l**) A GUS stain is present in the seedling roots and root tips. c, Cortex; cl, Carpel; e, Epidermis; mn, Minor Vein; mj, major vein r, Replum; p, Phloem; x, Xylem.

The public database, Arabidopsis eFP Browser (At-TAX), showed the LAT5/PUT5 expression in leaf, stem, flower, whole seedlings, and shoot apex. The Arabidopsis eFP Browser (tissue-specific) showed the LAT5/PUT5 expression in the leaf mesophyll and guard cells and stem epidermis (http://bbc.botany.utoronto.ca/efp/). Organ-specific quantitative RT-PCR was performed to see the presence and relative abundance of the LAT5/PUT5 transcripts in various organs such as root, stem, rosette leaf, senescing leaf, cauline leaf, flower, and silique. The LAT5/PUT5 transcripts were detected in all organs with relatively higher abundance in the leaves (Figure 2a).

Transgenic Arabidopsis plants expressing P_LAT5_: GUS were studied to elucidate the tissue-specific expression of the LAT5/PUT5. In these plants, GUS activity was detected in almost all organs with a pattern along the vasculature indicating expression in the vascular tissues (Figure 2b–j). In cross-sections of a rosette leaf, GUS activity was observed in the leaf mesophyll cells and minor veins (Figure 2d). Stem cross-sections showed GUS activity specifically in the phloem tissues (Figure 2f, g). In the flower, a GUS stain was present in the sepal and stamen filament (Figure 2h). In siliques, a GUS stain was present in the fruit carpel with a pattern along the vasculature (Figure 2i). Cross sectioning of a developing silique showed a GUS stain in the vascular tissues within the replum and the secondary vascular tissues in the carpel (Figure 2j). In mature plants, GUS activity was detected in the surface of primary and lateral roots (Figure 2k). At seedling stage, GUS activity was detected in roots and root tips (Figure 2l).

### 2.3. The Mutant Lat5-1 Shows Increased Sensitivity to Exogenous Leucine

**Figure 3:**
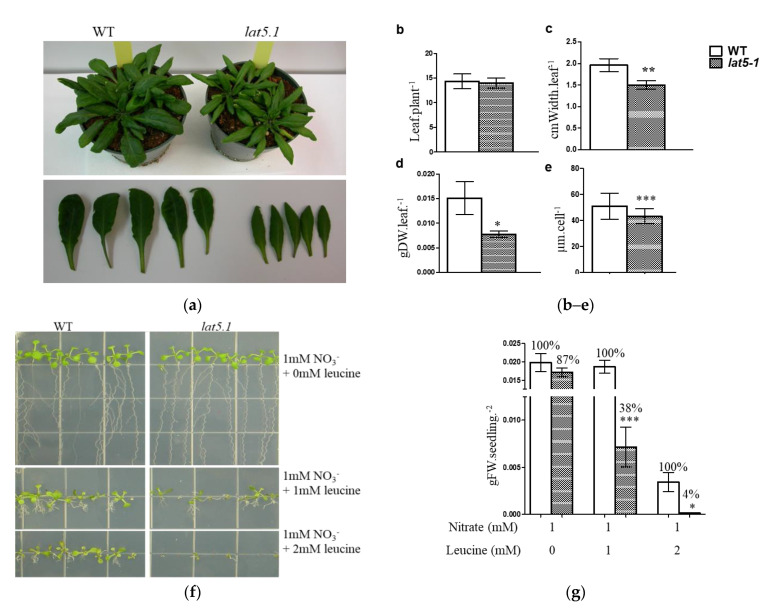
Phenotypic analysis of the *lat5-1* on soil and plate culture. (**a**) Visual presentation of the *lat5-1* showing reduced leaf growth compared to the WT; (**b**) the homozygous *lat5-1* mutant plants show no difference in the total number of rosette leaves; (**c**) but show reduced leaf size, measured by the leaf width; (**d**) and reduced biomass, compared to the WT under normal growth condition on fertilized soil. Photos were taken when the plants were five weeks old. For leaf biomass, leaves were cut off and dried at 70 °C for two hours before being weighed. The error bar represents the standard deviation of five replicates. (**e**) Leaf mesophyll cell protoplasts in the *lat5-1* are smaller compared to the WT. The error bar represents the standard deviation of the diameter of 100 cells from three individual plant protoplast cultures. (**f**) The mutant *lat5-1* seedlings are more sensitive to exogenous leucine compared to the WT. Variable concentrations of L-leucine were added to a nitrogen-free growth medium with 1 mmol·L^−1^ nitrate as the source of nitrogen. (**g**) A quantitative representation shows significantly increased sensitivity of the *lat5-1* seedlings to exogenous leucine compared to the WT, measured by the reduction in fresh weight of biomass. The relative percentage of growth was calculated assuming the biomass obtained by the corresponding WT as 100%. The error bar represents the standard deviation of three to five biological replications with two seedlings per replication. Photos were taken when the seedlings were two-weeks old. Each growth condition had three or more plate replications. Each growth experiment was repeated at least twice with similar results. * *p* < 0.05; ** *p* < 0.01; *** *p* < 0.001. DW, Dry weight; FW, Fresh weight; M, mol·L^−1^.

Homozygous *lat5-1* mutant plants showed reduced leaf size under normal growth conditions on the soil. This phenotype for the *lat5-1* was also reported by Ahmed et al., where they used the same T-DNA insertion line (Salk_007135), but called it as *put5* [18]. While the average number of leaves per plant in the *lat5-1* did not differ from the WT, the width of rosette leaves, the diameter of leaf protoplast, and leaf biomass in the *lat5-1* were significantly reduced compared to the wild type (WT) (Figure 3a–e).

Leucine was used to investigate the role of another member of the LAT family, LAT4/PAR1, in amino acid transport [11]. For the same reason, leucine was used to investigate the role of LAT5/PUT5 in this report. While the *lat5-1* showed a 13% growth reduction on 1 mM nitrate, it showed a significant 62% growth reduction compared to the WT in the presence of 1 mM leucine. In the presence of 2 mM leucine, the WT seedlings showed a noticeable reduction in growth while the *lat5-1* seeds barely germinated and showed a significant 96% growth reduction compared to the WT in the same growth medium (Figure 3f,g). Free amino acid analysis in these seedlings showed a significantly increased concentration of free amino acids in both the *lat5-1* and WT seedlings grown in the presence leucine, compared to those grown without leucine supplement in the growth medium (Table 1). In the presence of 1 mM leucine, both genotypes accumulated significantly higher concentration of leucine in the free amino acid pool, which increased further when leucine concentration in the growth medium increased to 2 mM. However, the higher concentration of the total free amino acids in *lat5-1* and WT seedlings, in the presence of leucine in the growth medium, can be attributed mostly to the higher concentration of leucine in these seedlings. If leucine is discounted, the change in the free amino acid pool in the *lat5-1* seedlings compared to the WT grown with or without leucine supplement is not significant (Table 1).

The homozygous *lat5-1* line also showed increased tolerance to salt and drought stress. In full strength Murashige and Skoog (MS) medium, the *lat5-1* showed increased salt tolerance compared to the WT in the presence of various concentrations of NaCl (Figure S1c). In soil, the *lat5-1* consistently showed increased drought tolerance compared to the WT. When watering was delayed, the WT plants started to wilt earlier than the corresponding *lat5-1* plants (data not shown).

### 2.4. The Lat5-1 Seedlings Accumulate Leucine When Treated in the Dark

**Figure 4:**
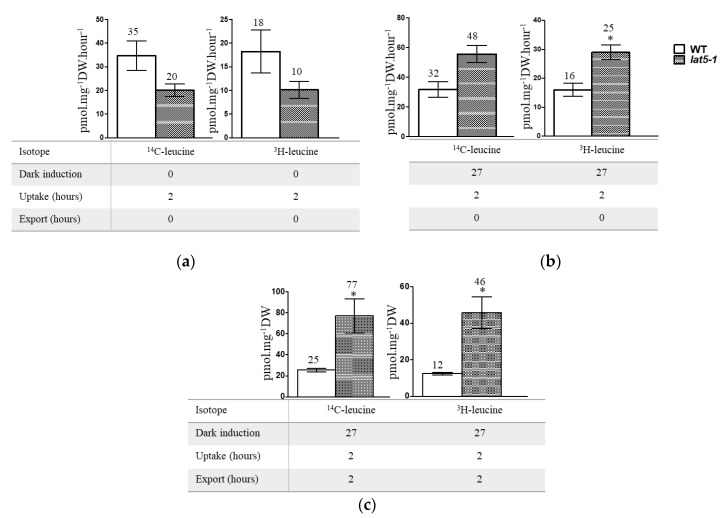
Radio-labelled leucine uptake study shows significantly increased accumulation of leucine in *lat5-1* compared to the WT when seedlings were treated in dark prior to the isotopic feeding and allowed to export. (**a**) Seedlings grown under normal light/dark regime show decreased accumulation of ^14^C- and ^3^H- in the *lat5-1* compared to the WT. (**b**) Seedlings, kept in dark for 27 h prior to the isotopic feeding, show increased accumulation of both ^14^C- and ^3^H- in the *lat5-1* compared to the WT. (**c**) Seedlings, kept in dark for 27 h prior to the isotopic feeding, show significantly increased accumulation of both ^14^C- and ^3^H- in the *lat5-1* compared to the WT when uptake was followed by export. Error bar represents standard deviation of three biological replications. * *p* < 0.05.

In a leucine uptake analysis, a mixture of ^14^C-leucine and ^3^H-leucine was used to trace leucine uptake and accumulation by the *lat5-1* seedlings, compared to the WT. Leucine was used due to its ability to cause hypersensitivity in the *lat5-1* seedlings (Figure 3f,g). The mutant *lat5-1* seedlings showed decreased accumulation of leucine compared to the WT, measured by the accumulation of ^14^C- and ^3^H-, when seedlings were grown under normal light/dark regime (Figure 4a). When seedlings were treated in the dark for 27 h before the isotopic feeding, the *lat5-1* accumulated increased leucine compared to the WT (Figure 4b). The difference in leucine accumulation between the WT and *lat5-1* genotypes were significant when seedlings were allowed to release the ^14^C- and ^3^H- back to the export medium for two hours following two hours of uptake (Figure 4c).

### 2.5. The lat5-1 Shows Increased Seed Weight and Nitrogen Content

**Figure 5:**
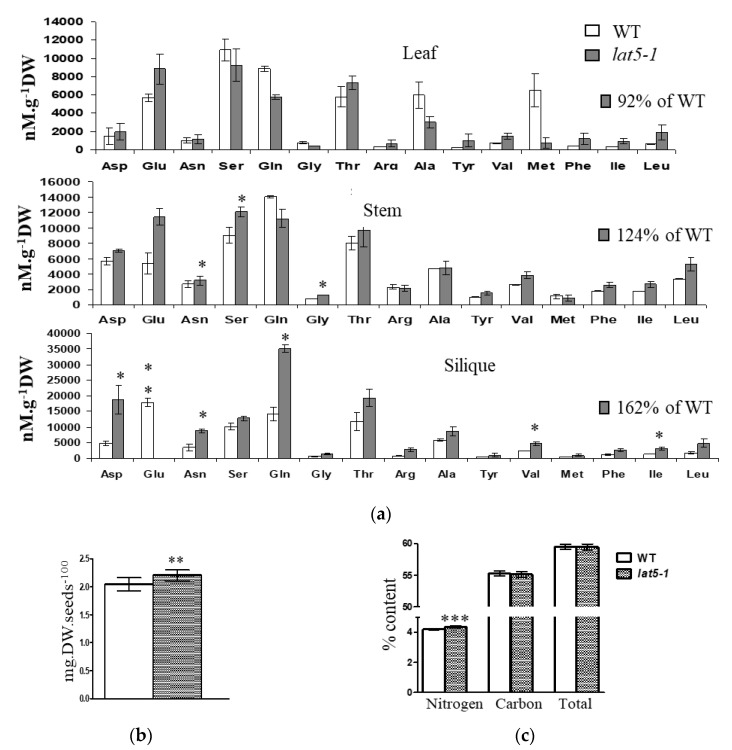
The *lat5-1* shows increased free amino acid content in the siliques and increased nitrogen content in the seeds, compared to the WT. (**a**) The total free amino acid concentration is increased in both stem and silique and decreased in the leaves in the *lat5-1* compared to the WT. The total free amino acids detected in the WT was arbitrarily taken as 100% to calculate the relative amount in the *lat5-1*. Error bar represents the standard deviation of three biological replications. (**b**) The seed weight is significantly increased in the *lat5-1*, compared to the WT. (**c**) The percent of nitrogen content is significantly increased in the *lat5-1*, compared to the WT. Error bar represents the standard deviation of nine replications from three individual plants with 100 seeds per replication. * *p*-value < 0.05; ** *p*-value < 0.01; *** *p*-value < 0.001.

An analysis of free amino acids was performed in the leaves, stems, and siliques of the *lat5-1* and WT plants. In general, the free amino acid profile in the *lat5-1* organs was altered in terms of both content and composition, compared to the WT. The total content of the 15 protein amino acids detected in the free amino acid pool decreased in the leaves (92%) but increased in the stems (124%) and siliques (162%) of the *lat5-1*, compared to the WT, with significantly increased concentration of specific amino acids such as asparagine and glutamine in the *lat5-1* siliques (Figure 5a).

The mutant *lat5-1* showed a 7% increased seed weight compared to the WT (Figure 5b). An analysis of total nitrogen content in the seeds showed that the *lat5-1* seeds contained 3% more nitrogen compared to the WT (Figure 5c).

## 3. Discussion

According to the in silico analysis, the LAT5/PUT5 is an integral membrane protein that has amino acid transporter and antiporter domains conserved in the sequence (Figure 1a,b) [11]. In human, the LATs conserve a cysteine residue in the second extra-cellular loop through which they form cys-cys di-sulfide bond with a glycoprotein subunit to function as exchangers [4,5,6,21]. The conserved cysteine residue in the LAT5/PUT5 (Table S1) did not position in any of the predicted intra- or extra-cellular loops (Figure 2b). However, the predicted Pox_P21 domain, conserved near the c-terminus in the LAT5/PUT5 (Figure 2b), is a characteristic domain in the Poxvirus P21 membrane protein that is localized in the inner membrane of the intra-cellular mature virus and acts as a membrane anchor for the externally located fusion protein [22]. This suggests that the LAT5/PUT5 may also have a binding partner. Further exploration of possible protein interactions within the Arabidopsis genome identified several proteins to have functional or physical associations with the LAT5/PUT5 protein (Figure 1c) [19,20]. These proteins may or may not have common functions, but these findings corroborate the possibility that the LAT5/PUT5 has binding partner(s). The LAT5/PUT5 was found to be localized to the endoplasmic reticulum (Figure S2) [18]. However, if the LAT5/PUT5, as suggested by the in silico analysis, binds with a partner through the conserved cysteine residue or the Pox_P21 domain at the c-terminus before tertiary folding and localization, having an epitope at this end may have hindered this protein to localize properly in these studies. We have interpreted the possible role of this transporter with caution, keeping in consideration that depending on the localization of the LAT5/PUT5 to the plasma membrane or organellar membrane, the deduced function of this transporter may vary. Previous studies implied that the LAT5/PUT5 mediates polyamine transport, but none of these reports demonstrated this with supporting data [17,18]. In this report, we studied the role of the LAT5/PUT5 in amino acid transport in the native physiological conditions.

The expression analysis suggests that LAT5/PUT5 is a widely expressed transporter (Figure 2). The mutant *lat5-1* showed reduced shoot growth on fertilized soil characterized by the smaller size of rosette leaves, reduced leaf biomass, and the smaller size of leaf protoplasts (Figure 3a–e). The growth reduction produced by the *lat5-1* mutant in the presence of both organic and inorganic nitrogen in soil could be due either to a reduced acquisition of amino-N from the soil, or a defect in internal nitrogen distribution, or a cumulative effect of both. The *lat5-1* seedlings were hypersensitive to the presence of leucine in the growth medium (Figure 3f,g). Analyzing the free amino acid pool in these seedlings showed that both the WT and *lat5-1* seedlings accumulated drastically higher concentration of leucine in the free amino acid pool which was correlated with the concentration of leucine in the growth medium, suggesting that the excessive leucine in the free amino acid pool in these seedlings were taken up from the growth medium (Table 1). This study suggested that the mutant *lat5-1* seedlings were not defective in taking up exogenous leucine. While the surplus leucine caused toxicity in both the *lat5-1* and WT seedlings, the *lat5-1* seedlings were presumably defective in mobilizing the excessive leucine and therefore exhibited hypersensitivity compared to the WT. In a radio-labelled leucine uptake study, the mutant *lat5-1* took up less leucine under normal conditions but accumulated more leucine when the plants were subject to an extended dark period (Figure 4a–c). These studies suggest that an altered physiological condition triggered by the prolonged dark period had a reverse impact on the role of LAT5/PUT5 in mediating leucine transport in these seedlings. These findings, along with the in silico prediction of antiporter domain in the LAT5/PUT5 protein (Figure 1a–c), and a strong indication that this transporter also plays a role in polyamine transport, indicate a transport activity of the LAT5/PUT5 different from a uniporter or symporter.

A transporter located in the phloem and mediates both amino acid and polyamine transport, as the LAT5/PUT5 appears to be, is likely involved in phloem loading and unloading of amino acids and polyamines and therefore should play an important role in both amino acids and polyamine distribution among the sources and sink tissues. However, in this report, we focused on its role in amino acid transport. The transport of amino acids from leaves to seeds occurs via the phloem. Loading amino acids from leaf mesophyll cells into the phloem minor vein may occur both symplastically and apoplastically. In many species including Arabidopsis, loading assimilates into the phloem occurs apoplastically, where amino acids are exported from mesophyll cells into the apoplasm, followed by active uptake into the sieve element-companion cell complex of the phloem [23]. Based on the expression analysis, the LAT5/PUT5 showed relatively higher abundance in the leaves and specific expression in the leaf mesophyll cells and phloem minor veins (Figure 2a–d), which suggests a role for this transporter in loading amino acids from the leaf mesophyll cells into the phloem. Amino acids, loaded into the phloem from source tissues in the leaf may undergo exchange between the phloem and xylem for upward translocation to the reproductive sink tissues [24]. The expression of the LAT5/PUT5 in the stem along the vascular transport strand (Figure 2f,g) indicates its involvement in an active exchange of amino acids between xylem and phloem. In the siliques, expression of the LAT5/PUT5 in the replum and in the secondary vasculature in the carpel (Figure 2h–j) suggests an important role for this transporter in translocating amino acids to/from the seeds. Based on a free amino acid analysis in the leaves, stems, and siliques, the *lat5-1* showed significantly increased concentration of specific amino acids including asparagine and glutamine—the two major nitrogen-carrier amino acids—in the siliques, compared to the WT (Figure 5a). Analysis of seed weight and nitrogen content showed increased seed weight and nitrogen content in the *lat5-1* compared to the WT (Figure 5b,c). The Arabidopsis Amino Acid Permease 2 (AAP2) showed a similar expression pattern in the stem and silique [25]. While the AAP2 knockout mutant showed a decrease in the leaf amino acid content, it also showed a decrease in the total seed nitrogen content [26]. This might be due to the transport mechanism of the AAP2 being different from the LAT5/PUT5. While the AAP2 is an H^+^-amino acid symporter [25,26], our studies indicated a transport activity of the LAT5/PUT5 that is unlike a symporter.

## 4. Materials and Methods

### 4.1. In Silico Analysis of the LAT5/PUT5 Protein Sequence

The LAT5/PUT5 amino acid sequence was taken from the Arabidopsis membrane protein database (http://aramemnon.botanik.uni-koeln.de/). Amino acid sequence alignment was carried out using online CLUSTAL 2.0.12 multiple sequence alignment tool (https://www.genome.jp/tools-bin/clustalw). The transmembrane domain prediction for the LAT5/PUT5 was performed using the TMHMM v.2.0 online prediction tool (http://www.cbs.dtu.dk/services/TMHMM/). The cartoon representation was produced using TMRPres2D (http://bioinformatics.biol.uoa-gr/TMRPres2D). Molecular weight and theoretical iso-electric point was calculated using an online bioinformatics tool (https://web.expasy.org/compute_pi/). Protein family domain search was performed in the Pfam and NCBI-CDD databases using online motif search tools (https://www.genome.jp/tools/motif/ and http://pfam.xfam.org/) [27]. The sequence identity search was carried out using the online PSI-BLASTp search in the NCBI non-redundant protein databank (https://blast.ncbi.nlm.nih.gov/Blast.cgi). Protein association searches were conducted using online tools STRING v.11.0 (https://string-preview.org/cgi/input?sessionId=bZTucx5gquj9&input_page_show_search=on) and BioGRID (https://thebiogrid.org/search.php?search=&organism=3702).

### 4.2. Isolation of Lat5-1 Knockout Mutant

The Columbia-0 ecotype of *Arabidopsis thaliana* was used in this research. A homozygous T-DNA insertion line (Salk_007135c) lacking transcripts of the LAT5/PUT5 was obtained from the SALK collections at the Arabidopsis Biological Resource Centre (Columbus, OH, US) [28]. After confirming the presence of T-DNA in the second exon and absence of functional transcripts (Figure S1a,b), this line was named *lat5-1* and used as a LAT5/PUT5 knockout mutant in the subsequent studies. A previous report also confirmed that this line lacked the LAT5/PUT5 transcripts and called it put5 [18].

The presence of the T-DNA in the LAT5/PUT5 (At3g19553) locus was primarily confirmed through a three-primer PCR screening scheme described in the Salk webpage (http://signal.salk.edu/). One primer specific to the left border sequence of T-DNA, LBb1 (5′-GCGTGGACCGCTTGCTGCAACT-3′), and two primers specific to the LAT5/PUT5 sequence (reverse primer 5′-TGACTCAGGTACAAACCCTCC-3′ and forward primer 5′-ATATTTGATTCCCCTCATGGC-3′) were used in the PCR screen. The position of the T-DNA in the second exon of LAT5/PUT5 was confirmed through aligning the sequence produced using the T-DNA left-border-specific primer. Reverse Transcriptase (RT)-PCR, using UTR-specific primers, confirmed the absence of functional size of LAT5/PUT5 transcripts in *lat5-1* (Figure S1b). For RT–PCR, total RNA was extracted, using the RNeasy Plant Mini Kit (QIAGEN, Darmstadt, Germany), from 100 mg of flash-frozen leaf tissues as per manufacturer’s instruction. To remove contaminating DNA, RNA was treated using Ambion’s DNA-free kit. The quantity and quality of RNA were checked by using Nanodrop (ThermoFisher, Waltham, MA, USA) and Bioanalyzer (Agilent 2100 Bioanalyzer, Agilent Technologies, Inc., Santa Clara, CA, USA). A one-step RT–PCR using both the LAT5/PUT5 and Actin2 specific primers in the same reaction vial was performed using SuperScript III One-Step RT–PCR System with Platinum Taq High Fidelity (Catalog No. 12574-030, Invitrogen, Carlsbad, CA, USA). Actin2 was used as a technical control and WT plant tissues were used as a positive control. The reactions were set up as per manufacturer’s instruction and the program for the thermal cycler was as follows: cDNA synthesis at 55 °C for 30 min, pre-denature at 94 °C for 2 min, 40 cycles of denature at 94 °C for 15 s, annealing at 55 °C for 30 s, extension at 68 °C for 1 min, final extension at 68 °C for 5 min.

### 4.3. Plant Growth and Data Analysis

All plants (unless otherwise mentioned) were grown on well-aerated fertilized soil in a growth chamber under a 16 h light–8 h dark regime under ~170 µE·m^−2^·s^−1^ light intensity at 23 °C daytime temperature. Growth condition and soil type were the same as described in Begam and Good, (2017) [11]. For phenotypic screening in the presence of leucine, nitrogen-free half-strength MS medium [28] was prepared using stock solutions of macronutrients, micronutrients, and vitamin mixture at a molar concentration described in the commercially available MS basal salt mixture (Catalog No. ICN2623120; Fisher Scientific, ON, Canada) without ammonium nitrate. To obtain the desired nitrogen concentration (1 mmol·L^−1^ nitrate and 1 mmol·L^−1^ or 2 mmol·L^−1^ leucine, Figure 3f), potassium nitrate and L-leucine (Sigma–Aldrich, Oakville, ON, Canada) were added to the MS-agar medium. For each growth condition, both genotypes were grown on the same plate with 3–5 plate replications. Sterile seeds were plated on MS agar medium and stratified by storing the plates at 4 °C temperature for three days and grown under 16 h light–8 h dark regime at 23 °C daytime temperature.

Data analysis and significance tests for all studies were carried out using PRISMv.5. A two-way T-test was performed for the statistical significance.

### 4.4. Expression Analysis

For the organ-specific quantitative RT-PCR, RNA was extracted from roots, stems, rosette leaves, cauline leaves, senescing leaves, flowers, siliques, and whole plant tissues separately. Total RNA was extracted, using the RNeasy Plant Mini Kit (Qiagen, Darmstadt, Germany) as described above, from approximately 100 mg (fresh weight) of flash-frozen tissues of each organ. All samples were adjusted to a final concentration of 50 ng·µL^−1^. For each sample, 2.5 µg of total RNA was used in the cDNA synthesis with oligo-dT and random primers (Invitrogen). The SuperScript II Reverse Transcriptase (Invitrogen, Carlsbad, CA, USA) was used in the reverse transcription for 50 min at 42 °C. The TaqMan Gene Expression Assay (assay ID: At02254880_gI for LAT5/PUT5; assay ID: At02335270_gH for Actin2) was used in PCR amplification. Actin2 was used as an endogenous control. The PCR program was as follows: 95 °C for 10 min followed by 40 cycles of 95 °C for 15 s and 60 °C for one minute. The relative quantitation was calculated using a 7500 Fast System SDS software (Applied Biosystems, Waltham, MA, USA). For data analysis, the average expression level in the whole plant tissues was used as a calibrator. The final graph was produced using RQ (Relative Quantitation) as an expression level and the mean difference between RQmax and RQmin as an error bar.

For the tissue-specific expression analysis, the promoter region of the LAT5/PUT5 (1288 bp upstream from the start codon) was PCR amplified using SalI and NcoI sites added to the 5′-end of forward (5′-GTCGACTGCGAGATATCGGACATCATA-3′) and reverse (5′-AACCATGGCTTAGAGCTGTTTTCATCG-3′) primers, respectively, and cloned into the pCAMBIA1305.1 vector (http://www.cambia.org/) between the SalI and NcoI site, replacing the CaMV35S promoter preceding the GusPlus coding region. The final expression vector with the P_LAT5_:GusPlus fusion was confirmed through PCR and restriction analysis. The transformation for producing a transgenic Arabidopsis line with P_LAT5_:GusPlus was carried out using a modified floral dip transformation protocol described by Clough and Bent [29]. The transformants were screened on a 0.5× MS medium containing 25 µg.mL^−1^ hygromycin and confirmed by PCR using GUS specific primers. The GUS histochemical assay was performed using a modified protocol described by Weigel and Glazebrook [30]. Samples were prepared by incubating three-week-old seedlings or mature plants in a GUS staining buffer (50 mM Na_2_HPO_4_, 2 mM Potassium ferricyanide, 2 mM Potassium ferrocyanide, 2 mM X-Gluc and 0.2% TritonX-100) for 30 min (seedlings) or six hours (mature plants) at 37 °C followed by 30 min of incubation in 70% ethanol at room temperature. Samples were fixed in FAA (50% ethanol, 10% Glacial acetic acid, 5% formaldehyde) overnight and stored in 70% ethanol at 4 °C until photographs were taken under a dissecting microscope. For the tissue-specific expression analysis, tissues were processed and embedded in wax. Thin cross-sections of 8 µm were produced using a microtome. Tissue sections were fixed on a glass slide by drying them at 39 °C for two days. Samples were de-waxed with toluene and counter-stained with safranin O prior to taking photographs under a light microscope.

### 4.5. In Planta Radio-Labelled Leucine Uptake Study

A ^14^C- and ^3^H-Leu uptake analysis was carried out using WT and homozygous *lat5-1* seedlings of approximately one-month-old, grown on fertilized soil under normal growth conditions. For dark treatment, seedlings in pots were kept in the dark for 27 h prior to isotopic feeding. Seedlings were removed from the soil and roots were rinsed with sterile water before use. ^14^C-leucine and ^3^H-leucine were added to the uptake medium (liquid MS without nitrogen and sucrose, pH 5.4) with cold leucine added to a final concentration of 10 µM·L^−1^, and used 15 mL uptake medium per seedling. Seedlings were removed from the uptake medium after two hours, and roots were rinsed three times in sterile water for a total of 10 min. When export was allowed following uptake, roots were rinsed with sterile water for five minutes after removing from the uptake medium and dipped in the export medium for another two hours. Half-strength liquid MS without nitrogen and sucrose was used as an export medium. Uptake was carried out under ambient light but export was carried out in the dark. During uptake, export or rinsing, only roots were dipped in the solutions to avoid surface contamination of radioactivity in the shoots. Roots and shoots were separated immediately after rinsing and stored directly in glass scintillation vials. Tissues were dried at 70 °C overnight and weighed. Samples were digested overnight at room temperature with 1 mL of 5% NaClO. Vials were kept open for at least 1 h under a fume hood before adding 5 mL HionicFluor (Ultima Gold XR, Perkin Elmer Inc., Waltham, MA, USA) to each vial. Radioactivity in the roots and shoots of the same plant were combined to obtain total radioactivity in each sample. Data were presented as radioactivity in pmol per milligram dry weight of shoots.

### 4.6. Analysis of Free Amino Acids through HPLC

For the HLPC analysis of free amino acids in seedlings grown in the presence of variable concentrations of leucine (Figure 3f and Table 1), three-week-old seedlings were used for amino acid extraction. For free amino acid analysis in the leaves, stems, and siliques, tissues were collected from mature plants. Samples were grounded in liquid nitrogen. For dry weight, tissue samples were lyophilized for 72 h before weighing. One and a half mL extraction buffer (methanol: chloroform: water at 65:25:10 ratio with 20 nmol.mL^−1^ internal standard (Norvaline and Sarcosine) was added to each sample. Samples were vortexed and centrifuged to separate the aqueous phase. Five hundred microliter of the supernatant was collected and 100 µL chloroform followed by 150 µL water was added to the supernatant. Samples were mixed and centrifuged. The upper aqueous phase (400 µL) was passed through a MILLEX GX 0.22 µm filter (UFC30GV0S) followed by a MILLIPORE BIOMAX 5KNMWL MEMBRANE filter (UFV5BCC00). The final filtrate was used in the HPLC analysis using a ZORBAX Rapid Resolution HT Eclipse Plus C18 1.8 µm 3.0 × 100 mm column. During extraction, the volume of reagents added and the volume of supernatant harvested were kept constant for all samples to maintain similar extraction efficiency across samples. An Agilent Technologies 1200 series automated liquid chromatography system was used to analyze the samples. Samples were derivatized pre-column with OPA (o-Phthalaldehyde) and FMOC (9-Fluorenylmethyl chloroformate). All chromatographs were processed in the Agilent Chemstation software suite. The area under each signal peak was calibrated by the standard calibration curve and normalized by the internal standard. All concentration data were back-calculated to the concentration in the samples in nmol per gram fresh weight or dry weight, as specified in Table 1 and Figure 5a. For each organ, the total amino acids in the WT was arbitrarily taken as 100%, and the %change in the *lat5-1* was calculated accordingly.

### 4.7. Analysis of Nitrogen Content in Mature Seeds

Mature and dry seeds harvested from soil-grown *lat5-1* and WT plants were weighed using a Mettler Toledo XP56 Delta Range Microbalance that had accurate readability up to 10 µg. For the analysis of total nitrogen and carbon content, nine sample replications were prepared from three individual plants from each genotype with 100 seeds per replication. A CE440 Elemental Analyser (Exeter Analytical Inc., North Chelmsford, MA, USA) was used to analyze the carbon (CO_2_), hydrogen (H_2_O), and nitrogen (N) content in the organic and inorganic compounds. The combustion of weighed samples was carried out in pure oxygen under static conditions at 975 °C. Helium was used to carry the combustion products through the analytical system. The products of combustion were passed over suitable reagents in the combustion tube to assure complete oxidation. In the reduction tube, oxides of N were converted to molecular N at 690 °C, and the residual oxygen was removed. Samples were passed through a thermal conductivity detector. The percentage of carbon and nitrogen in each sample replication was calculated by dividing the total mass of N or C by the weight of 100 seeds.

## 5. Conclusions

Our studies suggested that the LAT5/PUT5 plays a role in amino acid transport, while previous reports indicated that it is a polyamine transporter [17,18]. The possibility that the LAT5/PUT5 mediates transport of both amino acid and polyamine, its transport mechanism is bi-directional or antiporter, and it physically associates with other proteins for function, makes it a complex transporter of interest. The putative bi-directional or antiporter function of the LAT5/PUT5 suggests its role in amino acid and polyamine homeostasis. Free amino acids and polyamines are metabolites with an ability to influence gene expression, important biochemical pathways, and biotic and abiotic stress responses [31,32,33]. The loss of function of the LAT5/PUT5 has generated several phenotypic variations in the *lat5-1*, some of which are probably indirect impacts triggered by an altered amino acid/polyamine homeostasis. While these studies are insufficient to decipher the exact role of this transporter, these findings make the LAT5/PUT5 an important candidate to be studied further. Confirming its localization, transport mechanism and substrate specificity will allow a more accurate interpretation of its role in Arabidopsis.

## Figures and Tables

**Table 1:** Free amino acid analysis in the homozygous *lat5-1* seedlings grown on nitrate and leucine.

	1 mM NO_3_^−^ + 0 mM Leucine			1 mM NO_3_^−^ + 1 mM Leucine			1 mM NO_3_^−^ + 2 mM Leucine		
Amino acid (nmol.g^−1^ FW)	WT	*lat5-1*	%change in *lat5-1*	WT	*lat5-1*	%change in *lat5-1*	WT	*lat5-1*	%change in *lat5-1*
Asp	702	753	+7	825	866	+5	1116	1210	+8
Glu	2857	3492	+22	3764	3640	−3	3854	4867	+26
Asn	1233	1115	−10	523	496	−5	1899	2130	+12
Ser	1232	1332	+8	1350	1195	−11	2612	4437	+70
Gln	3336	2355	−29	1478	1457	−1	8278	7306	−12
His	47	36	−23	52	43	−18	160	318	+98
Gly	120	146	+21	167	157	−6	308	304	−1
Thr	397	348	−12	357	307	−14	498	610	+23
Arg	130	73	−44	387	338	−13	615	765	+24
Ala	535	522	−2	836	610	−27	861	1461	+70
Tyr	52	43	−17	123	102	−17	157	220	+40*
Val	3272	2697	−18	2653	1901	−28	3654	7545	+106
Met	56	50	−10	297	297	0	266	318	+19
Trp	50	34	−32	108	80	−26	108	197	+82
Phe	75	59	−21	265	252	−5	281	357	+27
Ile	159	109	−31	434	365	−16	723	1188	+64
Leu	159	131	−18	44,514	42,191	−5	64,664	76,066	+18
Lys	243	210	−14	453	329	−27	395	1037	+162*
Total	14,654	13,507	−8	58,586	54,625	−7	90,772	110,998	22
Total—Leu	14,495	13,376	−8	14,072	12,434	−12	26,108	34,932	+34
Total change WT vs. WT	100%			400%			619%		
Total change *lat5-1* vs. *lat5-1*		100%			404%			822%	

Each data is an average of three biological replications with two seedlings per replication. * *p*-value < 0.05.

## Supplementary Materials

The following are available online at https://www.mdpi.com/2223-7747/9/11/1519/s1, Figure S1. Isolation of homozygous *lat5-1* mutant line in Arabidopsis. (**a**) Position of the T-DNA in the second exon of the LAT5/PUT5 gene. Black and bold arrows indicate the positions of primers used in the three-primer PCR. Grey and thin arrows indicate the positions of primers used in RT-PCR. (**b**) RT-PCR shows the absence of LAT5/PUT5 transcript in the Salk_007135c line. (**c**) The homozygous *lat5-1* mutant shows increased salt tolerance compared to the WT on MS medium containing various concentrations of NaCl. Seedlings were grown on 1× MS medium (2% sucrose, 1% agar) with variable concentrations of NaCl to impose salt stress. Figure S2. Subcellular localization study of LAT5/PUT5. (**a**) Epi-fluorescence micrographs specific to Alexa-488, the fluorescent probe attached to the secondary anti-Myc antibody, show c-Myc tagged LAT5/PUT5 localized in the endoplasmic reticulum in a tobacco bright yellow-2 (BY-2) cell. (**b**) Epi-fluorescence micrographs specific to Alexa-594 attached to Concanavalin A, an ER specific marker stain, visualize the endoplasmic reticulum in the same cell as in (**a**) and (**c**). (**c**) Corresponding differential interference contrast (DIC) image for each cell. Cells were transformed transiently. Six hours after biolistic bombardment, the cells were fixed with formaldehyde, permeabilized with pectolyase and Triton X-100, and processed for immunofluorescence staining by incubating with mouse anti-Myc antibodies and Alex 488-conjugated goat anti-mouse antibodies. Cells were also incubated with Alexa 594-conjugated Concanavalin A (as a marker stain for endogenous endoplasmic reticulum).
