# Supplementary material for: The Arabidopsis L-Type Amino Acid Transporter 5 (LAT5/PUT5) Is Expressed in the Phloem and Alters Seed Nitrogen Content When Knocked Out
Source: Plants (Basel). 2020 Nov 9;9(11):1519. doi: 10.3390/plants9111519 (PMC7695346; doi:10.3390/plants9111519)
Supplement: Supplementary file 1 [file plants-09-01519-s001.pdf]

Table S1. Amino acid sequence alignment of the five Arabidopsis LATs. A cysteine residue conserved near the c-termini of all five members.

LAT3	----------------MGDYNMNEFAYGNLYDDDDG-DVGGSSKEGNNSIQKVSMLPLVF	43
LAT4	MQKRRIITVNPSASIEMSQYENNEVPYSSVGAD----EVPSSPPKATDKIRKVSMLPLVF	56
LAT1	MTELSSPNLDSASQKPRISTENPPPPPPHISIGVTTGDPATSPARTVNQIKKITVLPLVF	60
LAT5	----------------MGEEETIVN------------DENSSKPKPS---PKLTLLPLVF	29
LAT2	---------MAISEASKSSHELPVT------------TAESSGKKAT--AKKLTLIPLVF	37
	. : * . *::::****	
LAT3	LIFYEVSGGPFGAEGSVNAAG-PLLALLGFVIFPFIWCIPEALITAEMSTMFPINGGFVV	102
LAT4	LIFYEVSGGPFGVEDSVNAAG-PLLALLGFVIFPFIWSIPEALITAEMGTMYPENGGYVV	115
LAT1	LIFYEVSGGPFGIEDSVKAAG-PLLAIVGFIVFPFIWSIPEALITAEMGTMFPENGGYVV	119
LAT5	LIFYEVSGGPFGVEDSVKSGGGPLLALLGFLIFPLIWSIPEALVTAELATSFPENGGYVV	89
LAT2	LIYFEVAGGPFGEEPAVQAAG-PLLAILGFLIFPFIWSIPEALITAELSTAFPGNGGFVI	96
	**::**:***** * :*::.* ****::**::**:**.*****:***:.* :* ***:*:	
LAT3	WVSSALGTFWGFQVGWMKWLCGVIDNALYPVLFLDYLKSAVPALATGLPRVASILILTLL	162
LAT4	WVSSALGPFWGFQQGWMKWLSGVIDNALYPVLFLDYLKSGVPALGSGLPRVASILVLTIL	175
LAT1	WVTLAMGPYWGFQQGWVKWLSGVIDNALYPILFLDYLKSGIPILGSGIPRVAAILVLTVA	179
LAT5	WISSAFGPFWGFQEGFWKWFSGVMDNALYPVLFLDYLKHSFPVLDHVAARVPALLVITFS	149
LAT2	WAHRAFGSFVGSMMGSLKFLSGVINVASFPVLCVTYLDKLFPVLESGWPRNVCIFASTVV	156
	* *:*.: * * *::.**:: * :*:* : **. .* * .* .:: *.	
LAT3	LTYLNYRGLTIVGWTAVFMGVFSMLPFAVMSLVSIPQLEPSRWLVMD--LGNVNWNLYLN	220
LAT4	LTYLNYRGLTIVGWVAVLMGVFSILPFAVMGLISIPQLEPSRWLVMD--LGNVNWNLYLN	233
LAT1	LTYLNYRGLSIVGVAAVLLGVFSILPFVVMSFMSIPKLKPSRWLVVSKKMKGVNWSLYLN	239

LAT5	LTYLNYRGLHIVGFSAVVLAVFSLCPFVVMALLAVPNIRPKRWLFVD--TQKINWRGYFN 207
LAT2	LSFLNYTGLAIVGYAAVVLGLVSLSPFLVMSAMAIPKIKPHRWGSLG--TKKKDWNLYFN 214
	*::*** ** *** **.:.:.*: ** **. :::*::.* ** :. :* *:*
LAT3	TLLWNLNYWDSVSTLAGEVANPKKTLPKALCYGVIFVALSNFLPLLSGTGAIPLDRE-LW 279
LAT4	TLFWNLNYWDSISTLAGEVENPNHTLPKALFYGVILVACSYIFPLLAGIGAIPLERE-KW 292
LAT1	TLFWNLNYWDSVSTLTGEVENPSKTLPRALFYALLLVVFSYIFPVLTGTGAIALDQK-LW 298
LAT5	TMFWNLNYWDKASTLAGEVDRPGKTFPKALFGAVLLVMGSYLIPLMAGTGALSSSTSGEW 267
LAT2	TLFWNLNFWDNVSTLAGEVDEPQKTFPLALLIAVIFTCVAYLIPLFAVTGAVSVDQS-RW 273
	*::****:**. ***:*** .* :*:* ** .:::. : ::*::: **:. . . *
LAT3	TDGYLAEVAKAIGGGWLQLWVQAAAATSNMGMFLAEMSSDSFQLLGMAELGILPEIFAQR 339
LAT4	TDGYFSDVAKALGGAWLRWWVQAAAATSNMGMFIAEMSSDSFQLLGMAERGMLPEFFAKR 352
LAT1	TDGYFADIGKVIGGVWLGWWIQAAAATSNMGMFLAEMSSDSFQLLGMAERGMLPEVFAKR 358
LAT5	SDGYFAEVGMLIGGVWLKGWIQAAAAMSNLGLFEAEMSSDAFQLLGMSEIGMLPAFFAQR 327
LAT2	ENGFHAEAAEMIAGKWLKIWIEIGAVLSSIGLFEAQLSSSAYQLEGMAELGFLPKFFGVR 333
	:*: :: . :.* ** *:: .*. *.:*:* *::**.::** **:* *:** .*. *
LAT3	SRYG-TPLLGILFSASGVLLLSGLSFQEIIAAENLLYCGGMILEFIAFVRLRKKHPAASR 398
LAT4	SRYG-TPLLGILFSASGVVLLSWLSFQEIVAAENLLYCVGMILEFIAFVRMRMKHPAASR 411
LAT1	SRYR-TPWVGILFSASGVIILSWLSFQEIVAAENLLYCFGMVLEFITFVRLRMKYPAASR 417
LAT5	SKYG-TPTISILCSATGVIFLSWMSFQEIIEFLNFLYALGMLLEFAAFVKLRIKKPDLHR 386
LAT2	SKWFNTPWVGILISALMSLGLSYMNFTDIISSANFLYTLGMFLEFASFIWLRRKLPQLKR 393
	*:: ** :.** ** : ** :.* :*: *:** **.*** :*: :* * * *
LAT3	PYKIPVGTVGSILICVPPIVLICLVIVLSTIKVALVSFVMVVIGFLMKPCLNHMDGKKWV 458

LAT4	PYKIPIGTTGSILMCIPPTILICAVVALSSLKVAAVSIVMMIIGFLIHPLLNHMDRKRWV 471
LAT1	PFKIPVGVLGSVLMCIPPTVLIGVIMAFTNLKVALVSLAAIVIGLVLQPCLKQVEKKGWL 477
LAT5	PYRVPLNTFGVSMLCLPPSLLVILVMVLAAPKTFLISGVIIVLGFCLYPFLTLVKEKQWA 446
LAT2	PYRVPLKIPGLVVMCLIPSAFLVLILVFATKIVYLICGVMTIGAIGWYFLINYFRKTKIF 453
	:::: * ::*: * :: ::.:: . :. . : .: :. . .
LAT3	KFS---------VCSDLAEFQKENLDCEESLLR 482
LAT4	KFS---------ISSDLPDLQQQTREYEETLIR 495
LAT1	KFS---------TSSHLPNLME----------- 490
LAT5	RFIPEETRPVSGVSSESQLDEEHGDESAASLLP 479
LAT2	EFN--------EVIDDLDNNVNGEHPKVDDHNS 478
	.* .. :

LAT1:At5g05630; LAT2:At3g13620; LAT3:At1g31820; LAT4:At1g31830; LAT5:At3g19553

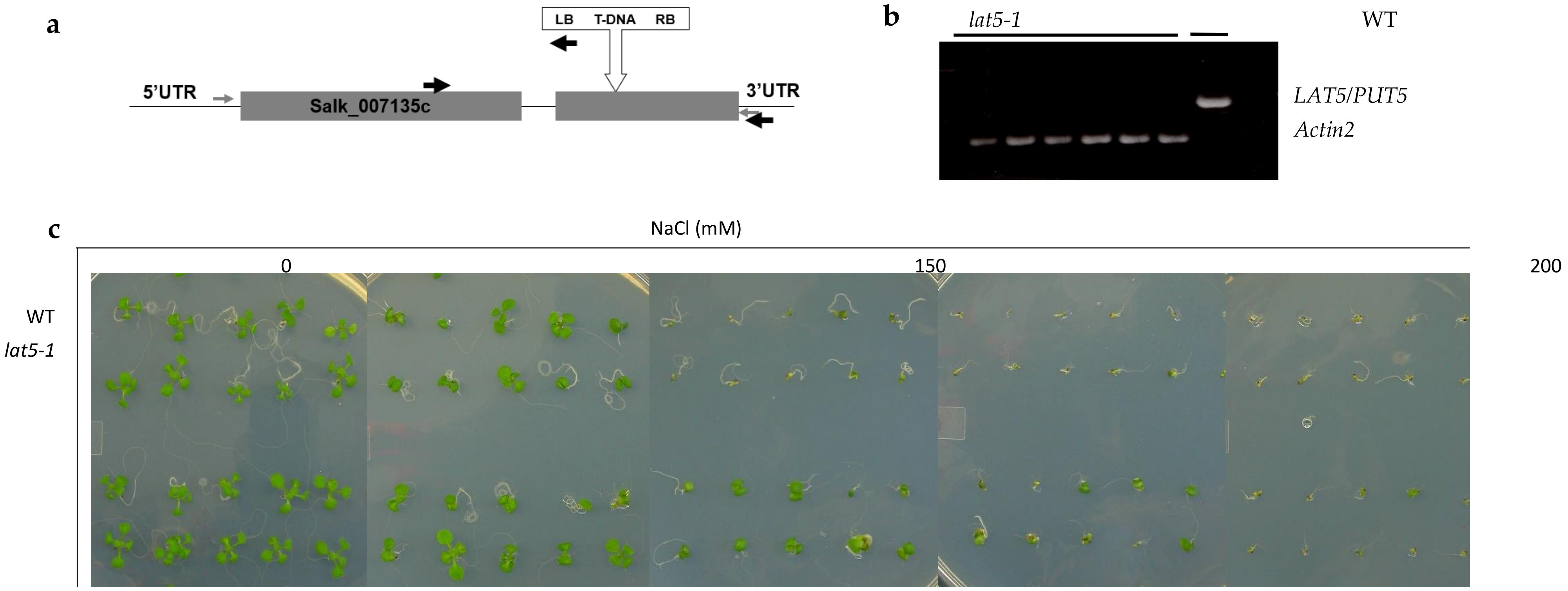

Figure S1. Isolation of the homozygous *lat5-1* mutant line in Arabidopsis. (**a**) Position of the T-DNA in the second exon of the LAT5/PUT5 gene. Black and bold arrows indicate the positions of primers used in the three-primer PCR. Grey and thin arrows indicate the positions of primers used in RT-PCR. (**b**) RT-PCR shows the absence of LAT5/PUT5 transcript in the Salk_007135c line. (**c**) The homozygous lat5-1 mutant shows increased salt tolerance compared to wild type on MS medium containing various concentrations of NaCl. Seedlings were grown on 1x MS medium (2% sucrose, 1% agar) with variable concentrations of NaCl to impose salt stress.

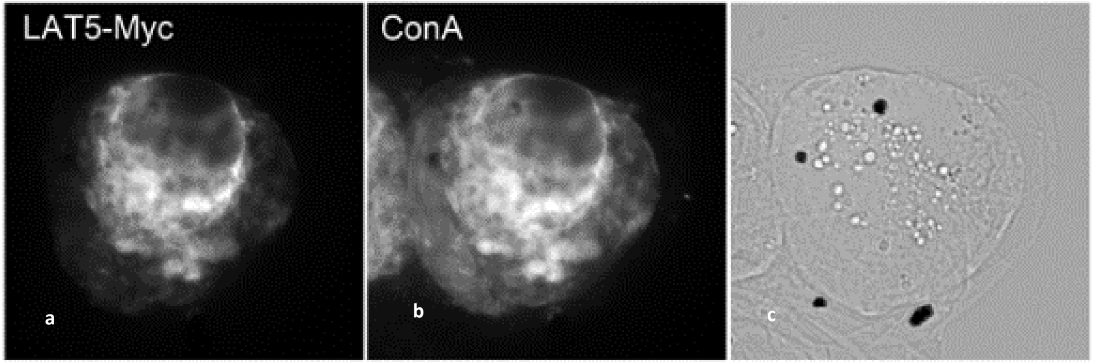

Figure S2. Subcellular localization study of the LAT5/PUT5. (**a**) Epi-fluorescence micrographs specific to Alexa-488, the fluorescent probe attached to the secondary anti-Myc antibody, show c-Myc tagged LAT5/PUT5 localized to the endoplasmic reticulum in a tobacco bright yellow-2 cell. (**b**) Epi-fluorescence micrographs specific to Alexa-594 attached to Concanavalin-A visualize the endoplasmic reticulum in the same cell as in (a and c). (**c**) Corresponding differential interference contrast image for each cell.

Cells were transformed transiently. Six hours after biolistic bombardment, the cells were fixed with formaldehyde, permeabilized with pectolyase and Triton X-100, and processed for immunofluorescence staining by incubating with mouse anti-Myc antibodies and Alex 488-conjugated goat anti-mouse antibodies. Cells were also incubated with Alexa 594-conjugated Concanavalin-A (as a marker stain for endogenous endoplasmic reticulum).
